# Physiological and Oxidative Stress in General and Spinal Anesthesia for Elective Cesarean Section in Women: Is There Any Difference?

**DOI:** 10.3390/life15081158

**Published:** 2025-07-22

**Authors:** Nemanja D. Dimic, Gorica D. Maric, Zorana S. Orescanin Dusic, Tanja M. Grahovac, Teodora F. Vidonja Uzelac, Marko D. Djuric, Irina B. Nenadic, Marina M. Bobos, Predrag D. Stevanovic, Sladjana J. Mihajlovic, Marina M. Stojanovic

**Affiliations:** 1 Clinic for Anesthesiology and Intensive Care, University Clinical Hospital Center “Dr Dragisa Misovic—Dedinje”, 11000 Belgrade, Serbia; drdjuric89@hotmail.com (M.D.D.); nenadicirina@gmail.com (I.B.N.); marinamorisan@gmail.com (M.M.B.); baticaster@gmail.com (P.D.S.); 2 Faculty of Medicine, University of Belgrade, 11000 Belgrade, Serbia; drsladjanamihajlovic@gmail.com (S.J.M.); marinailicstojanovic@gmail.com (M.M.S.); 3 Institute of Epidemiology, Faculty of Medicine, University of Belgrade, 11000 Belgrade, Serbia; goricamaric87@gmail.com; 4 Department of Physiology, Institute for Biological Research “Sinisa Stankovic”—National Institute of the Republic of Serbia, University of Belgrade, 11000 Belgrade, Serbia; zoranaor@ibiss.bg.ac.rs (Z.S.O.D.); tanja.grahovac@ibiss.bg.ac.rs (T.M.G.); teodora.vidonja@ibiss.bg.ac.rs (T.F.V.U.); 5 Department of Gynecology and Obstetrics, University Clinical Hospital Center “Dr Dragisa Misovic—Dedinje”, 11000 Belgrade, Serbia; 6 Center for Anesthesiology and Resuscitation, Clinical Center of Serbia, 11000 Belgrade, Serbia

**Keywords:** cesarean section, general anesthesia, spinal anesthesia, physiological stress, oxidative stress, biomarkers

## Abstract

This study evaluates the influence of general anesthesia (GA) and spinal anesthesia (SA) on physiological and oxidative stress in parturients undergoing elective cesarean section, one of the most frequently performed surgical procedures worldwide. A total of 101 pregnant women were included, categorized into GA (n = 51) and SA (n = 50) groups. Blood samples were collected at three time points: one hour before surgery (Measurement 1), at umbilical cord clamping (Measurement 2), and two hours post-surgery (Measurement 3). Biomarkers of oxidative stress, complete blood count, and levels of biochemical parameters were measured. In second and/or third measurement, biochemical blood analysis showed increased prolactin and cortisol levels, followed by spike of glucose and insulin in the GA group. However, levels of tri-iodothyronine were reduced in both groups in the third measurement. Glutathione S-transferase (GST) activity was increased in both groups in third measurement. The results showed increased concentrations of total SH groups and decreased concentrations of non-protein SH groups in the GA group during Measurement 2. Lymphocyte count was found to be predictor of GST levels. The results indicate more a pronounced endocrine response in GA group and speak in favor of spinal anesthesia. Both kinds of anesthesia are equally safe in terms of the oxidative status of the tissue.

## 1. Introduction

Cesarean section is one of the most performed surgeries worldwide, with increasing rates exceeding 50% in some countries, despite the World Health Organization’s recommendation of 10–15% [1].

Although, compared to a vaginal birth, cesarean section carries an increased risk for short-term and long-term complications, it is sometimes the safest or only way for women to give birth [2,3]. Spinal (regional) neuraxial anesthesia is currently recommended as the gold standard for cesarean delivery, as the mortality rate of cesarean section under general anesthesia is 16.7 times higher than that of spinal anesthesia [4]. The proposed benefits of regional techniques over general anesthesia include an earlier return of gut function, reduced incidence of pulmonary dysfunction, reduced inflammatory response to surgery, and beneficial effects on the coagulation system [5]. However, in pregnant women with urgent conditions such as abruption of the placenta, prolapse of the umbilical cord, antenatal bleeding of the placenta, and worsening of the general condition of the fetus, it is recommended to perform a cesarean section under general anesthesia [6]. Besides emergency cases, cesarean sections are also performed under general anesthesia in situations that are considered “inevitable and necessary,” such as, e.g., the refusal of a pregnant woman, contraindications for regional neuraxial anesthesia, or the need for rapid induction of anesthesia and hemodynamic stability [7]. With the development of technology, the incidence of complications arising as a result of general anesthesia, as well as the mortality rate of pregnant women giving birth by cesarean section under general anesthesia, has been significantly reduced [8].

In addition to the known advantages and disadvantages of general and spinal anesthesia during a cesarean section [9], the effect of anesthesia in terms of the physiological and oxidative stress of the parturient has yet to be sufficiently investigated. The stress response to surgery is a complex neuroendocrine–metabolic and inflammatory–immune process characterized by increased secretion of pituitary hormones and activation of the sympathetic nervous system [5]. Surgical stress may impair metabolism, thereby negatively affecting the body’s ability to grow, heal, maintain homeostasis, or adapt to the patient’s surroundings [10]. Major stress hormones include cortisol and catecholamines. Cortisol is a primary stress hormone released by the adrenal glands, often followed by the release of prolactin as part of the body’s coping mechanisms during stressful situations [11]. Besides regulating stress responses, cortisol also regulates metabolism, inflammatory responses, and immune functioning. It promotes protein breakdown and gluconeogenesis in the liver, resulting in increased blood glucose levels [11]. During childbirth, both vaginal and cesarean section, there is the expected formation of free reactive oxygen species, the consumption of antioxidants, and the occurrence of oxidative stress in the mother and fetus. Oxidative stress has proven to disrupt metabolic pathways, damage organ functions, and lead to severe complications [12]. The human body has systems for maintaining balance between antioxidants and systems that generate reactive oxygen species, which consist of both enzymatic and non-enzymatic processes [13]. Enzymatic antioxidant defenses include superoxide dismutase (SOD), glutathione peroxidase (GSH-Px), glutathione reductase (GR), and catalase (CAT) [14]. Previous research has reported conflicting results regarding the effect of anesthesia type on oxidative stress in surgical patients [15]. Therefore, our research aims to investigate the impact of general and spinal anesthesia on the physiological and oxidative stress responses of parturients following cesarean section. Additionally, our aim was to identify potential predictors of changes in oxidative stress enzyme activities during cesarean sections, considering the type of anesthesia administered.

## 2. Material and Methods

### 2.1. Study Design

A prospective cohort study was conducted at the Clinical Hospital Center “Dr Dragisa Misovic”, Belgrade, Serbia. A total of 101 pregnant women who underwent an elective caesarean section were included in this study in the period from 1 March to 1 July 2023. All participants gave their written informed consent. This study was approved by the Ethics Committee of the Faculty of Medicine, University of Belgrade (approval number: 17/I-4) and the Ethics Committee/Institutional Board of the Clinical Hospital Center “Dr Dragisa Misovic” (approval number: 01-3903/9-2022).

### 2.2. Selection of Participants

All pregnant women categorized as class II according to the classification of the American Association of Anesthesiologists during the study period were considered eligible for inclusion in this study [16]. Exclusion criteria were emergency cesarean section, premature birth, and pregnant women categorized as class III according to the American Association of Anesthesiologists classification. Participants in whom, according to clinical indications, the cesarean section was performed with the application of general anesthesia were included in the GA group, and participants in whom the cesarean section was performed using spinal anesthesia were included in the SA group. The type of anesthesia used in pregnant women is determined based on clinical indications and contraindications for the application of general and regional anesthesia.

### 2.3. Procedures

Data on demographic and pregnancy characteristics were collected from participants’ medical histories and by means of a questionnaire designed for study purposes. In both groups, blood samples for laboratory analysis were taken from each participant at three different time points: one hour before the start of the cesarean section (Measurement 1), at the moment of clamping the umbilical cord (Measurement 2), and two hours after the end of the cesarean section (Measurement 3). Whole blood samples were taken in heparinized vacutainers. Erythrocytes and plasma were immediately separated by centrifugation (5 min, 3000 rpm, 20 °C). Aliquots of three-times-washed erythrocytes with saline (0.9% ww^−1^) were lysed in ice-cold distilled water. The assessment of oxidative stress level was conducted by measuring the activity of the enzymes SOD, CAT, GSH-Px, GR, and glutathione S transferase (GST) and the concentrations of total (protein and non-protein) and non-protein sulfhydryl groups (SH). The activities of antioxidant enzymes were measured in lysates spectrophotometrically (U/mg Hb), while the activity of GST (U/mL plasma) and concentrations of total and non-protein SH groups were measured in plasma.

All antioxidant enzyme activities and hemoglobin were measured as described previously [17,18], as and we performed measurement of the GST activity and the concentrations of total and non-protein SH groups [19].

In addition to oxidative stress enzymes, the complete blood count and levels of biochemical parameters associated with stress were determined: the fasting blood glucose level, prolactin, cortisol, insulin, thyroid hormones, triglycerides, hemoglobin, hematocrit, fibrinogen, creatinine, albumins, C-reactive protein, electrolytes, alanine transaminase, aspartate transferase, and lipase.

The patients in both groups did not consume food for at least 6 h and water/liquids for at least 2 h before the beginning of the cesarean section. All cesarean sections were performed in morning hours from 8 a.m. to 12 a.m. Prior to the cesarean section, in each patient’s left arm, an intravenous cannula (18G) was placed, through which participants preoperatively received about 10 mL/kg of lactated Ringer’s crystalloid solution, 40 mg of pantoprazole (Takeda GMBH, Germany) intravenously, and 4 mg of ondansetron (Vianex s.a.—Plant A, Pallini, Greece) intravenously. For patients in the GA group, introduction to general anesthesia was performed using 2–2.5 mg/kg of propofol (Fresenius Kabi, Graz, Austria) and 0.6 mL/kg of rocuronium (Demo SA, Kryoneri, Greece). Anesthesia was maintained using a gas mixture of Sevoflurane (Baxter Healthcare Coproration, Deerfield, IL, USA) (1–1.5 Vol%), air, and oxygen (50%), with a flow rate of 2 L/min. After clamping the umbilical cord, the patients were prescribed 3–5 mcg/kg of fentanyl (Panpharma, Trittau, Germany). Upon awakening from general anesthesia, all patients were prescribed neostigmine (Cooper S.A. Pharmaceuticals, Athens, Greece) (0.08 mg/kg) and atropine (Sopharma AD, Sofia, Bulgaria) (1 mg intravenously).

In the SA group, the patients received spinal neuraxial anesthesia in a sitting position. Respecting the conditions of asepsis and antisepsis, interspinous space was initially identified at the L2–L3 level; then, the 25G needle was placed in the subarachnoid space, which was identified by the appearance of cerebrospinal fluid. After that, 1.8 to 2 mL of 0.5% levobupivacaine (Fresenius Kabi, Graz, Austria) was applied to the mentioned space. The motor and sensory blocks were tested using the prick and pinch test. After the cesarean section, all patients were transferred to the intensive care unit, where their vital parameters were monitored.

### 2.4. Statistical Analysis

Sample size was calculated using a formula for cohort studies, and according to this equation, assuming a power of the study of 80% and a type I error of 0.05, it was revealed that the required sample size was at least 35 participants per group. The data analysis used descriptive and inferential statistical methods. For the description of demographics and clinical characteristics, the means and standard deviations were calculated. For nominal and categorical variables, frequencies with percentages were presented. Changes over time in parameters of oxidative stress biomarkers in relation to the type of anesthesia were investigated using one-way ANOVA, followed by Tukey’s Honestly Significant Difference post hoc test (significance: *p* < 0.05). The means and standard deviations were calculated. To model the relationship of dependent variables in repeated measurements with potential predictors, linear mixed models were used. Multivariate regression models included predictors from univariate analyzes with *p* < 0.05. All calculations were performed in Statistical Package for Social Sciences, version 20.0, while the mixed effects model univariate and multivariate analyses were performed in R-4.3.3 software (The R Foundation for Statistical Computing, Vienna, Austria). The significance level was set at 0.05.

## 3. Results

### 3.1. Study Sample

The demographic and clinical characteristics of the compared groups (GA and SA) and the overall study sample are shown in Table 1. The participants in the GA and SA groups were comparable in terms of all baseline demographic and clinical characteristics (*p* > 0.05), indicating the homogeneity of the groups. The mean age of the participants was 33.1 ± 0.7 years for GA and 33.2 ± 0.6 years for SA group. Most of them lived in a house, had a body mass index (BMI) classified as overweight, were non-smokers, did not consume alcohol, had a low level of physical activity, had no comorbidities, had previous surgical procedures, and were in the 39th gestational week.

### 3.2. Laboratory Findings/Biochemical Parameters Associated with Stress

**Figure 1:**
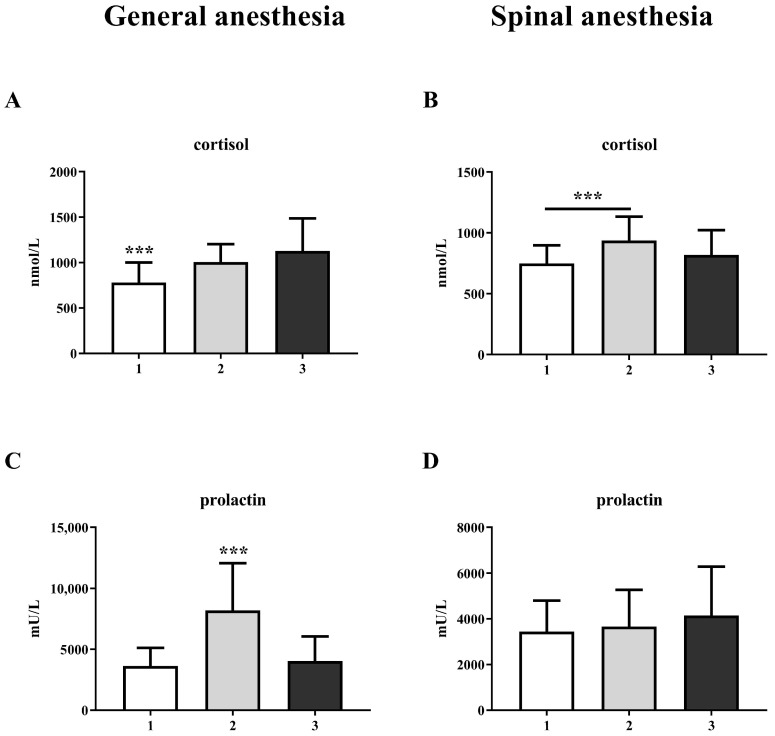
Blood cortisol and prolactin levels in general anesthesia (GA) and spinal anesthesia (SA) groups: (**A**)—GA cortisol level; (**B**)—SA cortisol level; (**C**)—GA prolactin level; (**D**)—SA prolactin level. The results are expressed as the mean (SD). Statistical significance was calculated by one-way ANOVA and a post hoc test compared by Tukey’s HSD test. ***—*p* < 0.001.

A comparison of the laboratory results for three measurements show significant increases in cortisol and prolactin levels in the GA group (Figure 1A,C). The increase in prolactin is significant at the second measurement, during surgery, and returns to control levels after 2 h (one-way ANOVA, F = 44.56; *p* < 0.001), while the cortisol level remains elevated 2 h after surgery (one-way ANOVA, F = 21.89; *p* < 0.001). In the SA group, prolactin does not change (Figure 1D), while cortisol levels increase only at the second measurement (one-way ANOVA, F = 13.36; *p* < 0.001) (Figure 1B).

**Figure 2:**
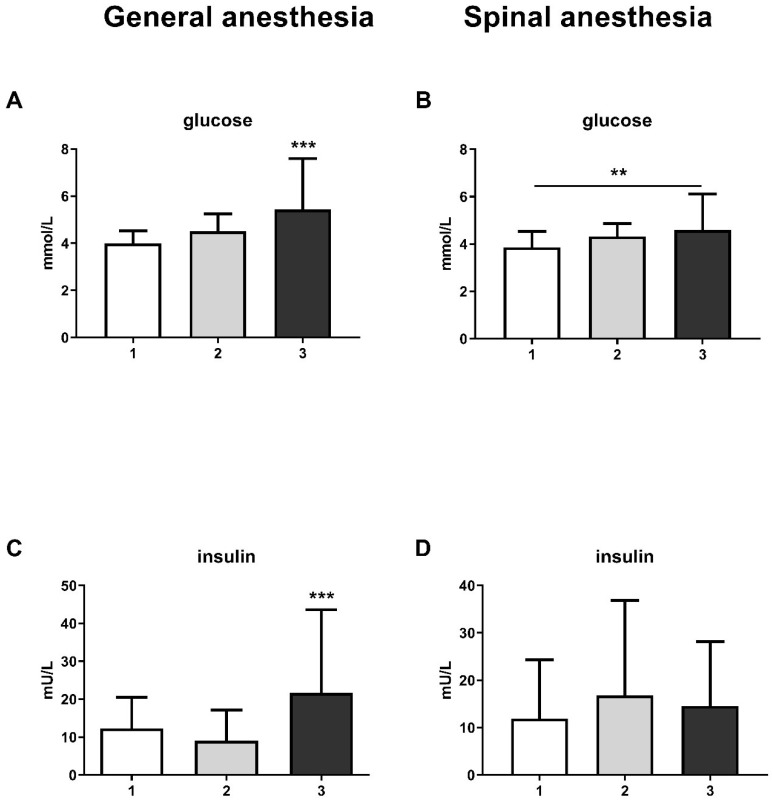
Blood glucose and insulin levels in general anesthesia (GA) and spinal anesthesia (SA): (**A**)—GA glucose level; (**B**)—SA glucose level; (**C**)—GA insulin level; (**D**)—SA insulin level. The results are expressed as the mean (SD). Statistical significance was calculated by one-way ANOVA and a post hoc test compared by Tukey’s HSD test. ***—*p* < 0.001; **—*p* < 0.01.

The results also show an increase in glucose levels at the third measurement, both in the GA group (one-way ANOVA, F = 14.28; *p* < 0.001) and in the SA group (one-way ANOVA, F = 6.53; *p* < 0.01) (Figure 2A and Figure 2B, respectively). This change was followed by an increase in insulin levels in the GA group at the third measurement (one-way ANOVA, F = 10.53; *p* < 0.001), while there was no change in the SA group (Figure 2C,D).

**Figure 3:**
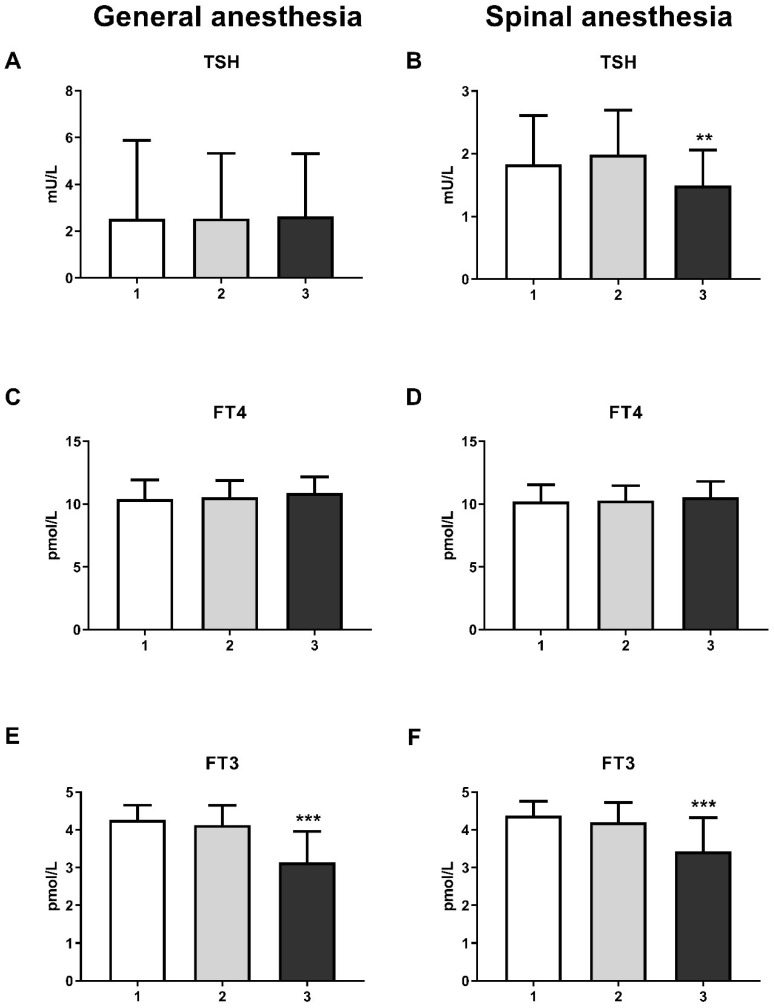
Blood thyroid hormones levels in general anesthesia (GA) and spinal anesthesia (SA): (**A**)—GA TSH level; (**B**)—SA TSH level; (**C**)—GA FT4 level; (**D**)—SA FT4 level; (**E**)—GA FT3 level; (**F**)—SA FT3 level. The results are expressed as the mean (SD). Statistical significance was calculated by one-way ANOVA and a post hoc test compared by Tukey’s HSD test. ***—*p* < 0.001; **—*p* < 0.01.

The level of thyroid hormones, more specifically tri-iodothyronine (FT3), was reduced at the third measurement in both the GA (one-way ANOVA, F = 51.6; *p* < 0.001) and SA groups (one-way ANOVA, F = 31.01; *p* < 0.001) (Figure 3E,F). It is interesting to note that the thyroid-stimulating hormone (TSH) level was only significantly reduced in the SA group (Figure 3B) at the third measurement (one-way ANOVA, F = 6.59; *p* < 0.01). The level of thyroxine (FT4) remain unchanged in both groups (Figure 3C,D).

### 3.3. Analysis of Changes in Biomarkers of Oxidative Stress over Three Measurements Between Groups

**Figure 4:**
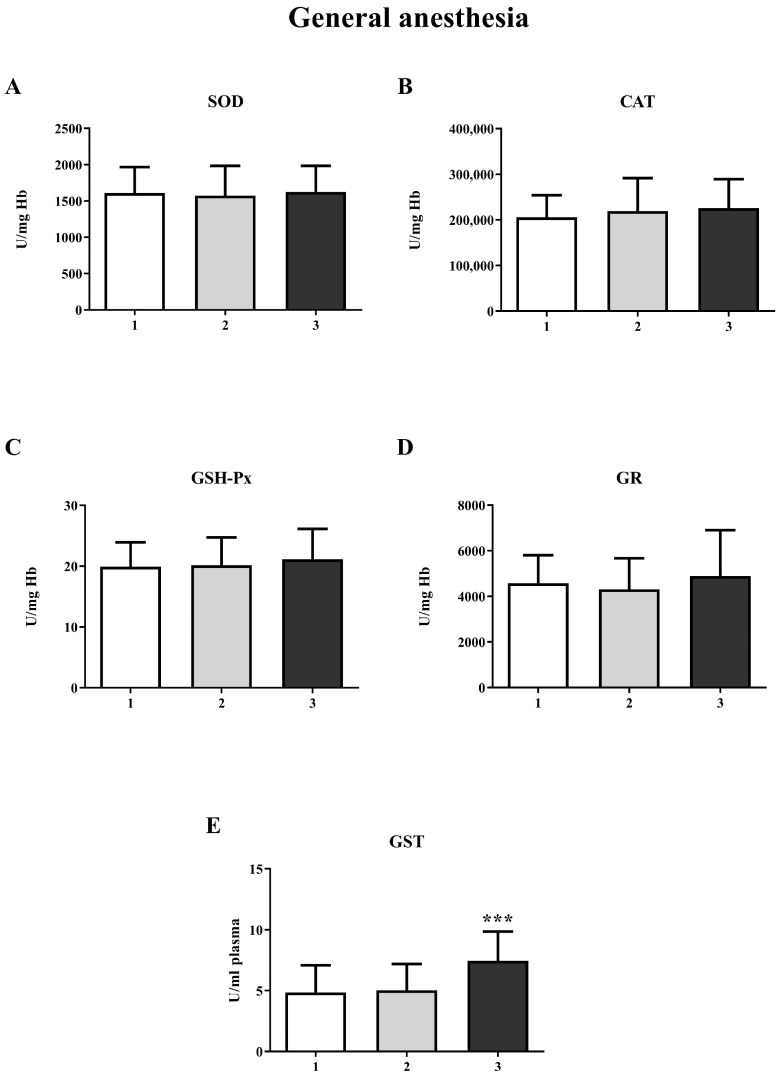
Activity of antioxidant enzymes at three different time points in general anesthesia (GA): (**A**)—superoxide dismutase (SOD); (**B**)—catalase (CAT); (**C**)—glutathione peroxidase (GSH-Px); (**D**)—glutathione reductase (GR); (**E**)—glutathione S-transferase (GST). The results are expressed as the mean (SD). Statistical significance was calculated by one-way ANOVA and a post hoc test compared by Tukey’s HSD test. ***—*p* < 0.001.

**Figure 5:**
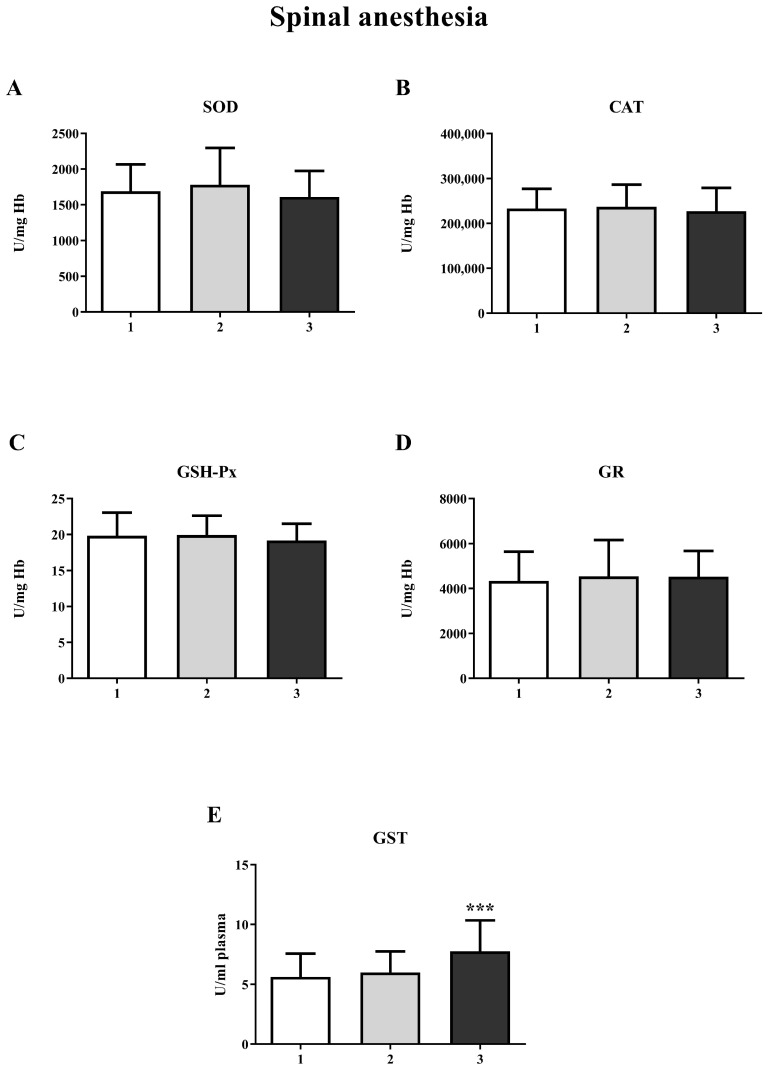
Activity of antioxidant enzymes at three different time points in spinal anesthesia (SA): (**A**)—superoxide dismutase (SOD); (**B**)—catalase (CAT); (**C**)—glutathione peroxidase (GSH-Px); (**D**)—glutathione reductase (GR); (**E**)—glutathione S-transferase (GST). The results are expressed as the mean (SD). Statistical significance was calculated by one-way ANOVA and a post hoc test compared by Tukey’s HSD test. ***—*p* < 0.001.

**Figure 6:**
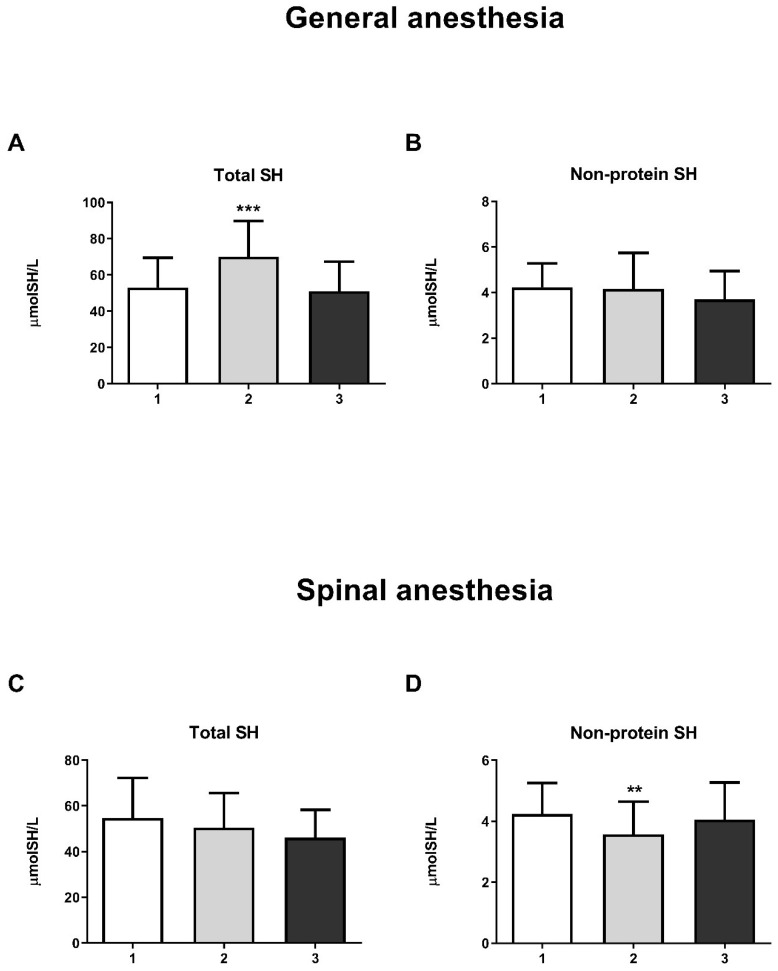
Concentrations of total SH groups and non-protein SH groups in general anesthesia (GA) and spinal anesthesia (SA): (**A**)—Concentration of total SH groups in the GA group; (**B**)—Concentration of non-protein SH groups in the GA group; (**C**)—Concentration of total SH groups in the SA group; (**D**)—Concentration of non-protein SH groups in the SA group. The results are expressed as the mean (SD). Statistical significance was calculated by one-way ANOVA and a post hoc test compared by Tukey’s HSD test. ***—*p* < 0.001; **—*p* < 0.01.

The results showed a significant increase in GST activity in the third measurement in both the GA (one-way ANOVA, F = 20.63; *p* < 0.001) and SA groups (one-way ANOVA, F = 14.16; *p* < 0.001) (Figure 4E and Figure 5E, respectively). For other biomarkers of oxidative stress (SOD, CAT, GR, and GSH-Px), there were no significant changes over time. A significant increase in the concentration of total SH groups was observed in the GA group (Figure 6A) during the second measurement (one-way ANOVA, F = 17.73, *p* < 0.001). However, a decrease in the non-protein SH group concentration was noticed in the second measurement in the SA group (one-way ANOVA, F = 4.77, *p* < 0.01) (Figure 6D).

### 3.4. Predictors of Changes in Biomarkers of Oxidative Stress over Three Measurements Between Groups

The results of the mixed effects model analysis are presented in Table 2. The following variables were singled out as independent contributors of change in oxidative stress parameters over three measurements: the lymphocyte count (*p* < 0.05) as a predictor of GST levels, alanine transaminase (*p* < 0.05) as a predictor of the GSH-Px level and obesity (*p* < 0.01), and the presence of thrombophilia (*p* < 0.01) and the leukocyte count (*p* < 0.05) as predictors of GR levels. Biochemical parameters related to potential predictors of oxidative stress are presented in Table S1.

## 4. Discussion

To our knowledge, there are not many studies that have investigated the effects of physiological and oxidative stress during the use of general anesthesia and spinal anesthesia for cesarean section.

Our results show an increase in cortisol and prolactin levels during cesarean section under both general and spinal anesthesia. The basal level of prolactin gradually increases during pregnancy, which is attributed to the stimulating effect of estrogen and the development of lactotropic hyperplasia. Before delivery, the prolactin level can increase more than 10-fold, reaching a peak of up to 200 ng/mL in the serum. Stress-induced prolactin release can also reach 2–3 times higher levels compared to baseline within a few minutes of exposure to stressors [20]. In abdominal aortic surgery, the combination of epidural anesthesia with general anesthesia reduces the increase in cortisol and urinary adrenaline concentrations during surgery compared to general anesthesia alone [21]. Our results are consistent with those of Mujagic et al. [22], who investigated the effects of intravenous general anesthesia with propofol–fentanyl and balanced anesthesia with isoflurane–fentanyl on circulating levels of stress hormones in patients undergoing elective low-abdominal surgery. They showed that mean serum concentrations of cortisol and prolactin were significantly higher during and shortly after surgery compared to controls, but lower in propofol–fentanyl-treated patients than in isoflurane–fentanyl-treated patients. The significantly higher increase in cortisol and prolactin in the GA group compared to the SA group in our study indicates a more pronounced endocrine response in patients treated under general anesthesia compared to spinal anesthesia.

A change in glucose and insulin levels accompanied the change in stress hormone levels in our study. Cortisol is known to increase blood glucose levels by causing the liver to release glycogen into the bloodstream [23]. Therefore, the pancreas produces more insulin to try to normalize these levels. On the other hand, prolactin has various functions that affect not only reproduction but also metabolism, osmoregulation, immune regulation, and behavior [20]. Prolactin affects insulin sensitivity and glucose metabolism, enhances hepatic insulin sensitivity, and disrupts immune function [24,25]. Extremely high prolactin levels increase insulin resistance and damage insulin secretory capacity in situations such as prolactinoma [26]. Considering the greater increase in glucose levels, followed by a significant increase in insulin levels, in the GA group, our results again support the use of spinal anesthesia in reducing stress. Additionally, Milosavljevic and colleagues have demonstrated that spinal anesthesia results in the suppression of serum cortisol and blood glucose concentrations compared to general anesthesia in patients undergoing elective abdominal, urological, and orthopedic surgery [27], confirming our assumption about the advantages of spinal anesthesia in surgical interventions.

The increase in cortisol also led to changes in thyroid hormone levels. Elevated cortisol is known to prevent the conversion of FT4 to FT3 [28]. This effect could be due to either a reduction in TSH concentration [29,30] or a slowing of FT4 turnover [31]. It is precisely the reduced level of FT3 that emerges from our results in both groups. In the SA group, this change was accompanied by a decrease in TSH levels.

Although we found no studies that examined the influence of anesthesia type on oxidative stress during cesarean sections, some studies have shown the influence of anesthesia on oxidative stress during other types of surgery. Aremu and his colleagues investigated the impact of general and spinal anesthesia on orthopedic patients. They concluded that there were significant changes in the concentration of malondialdehyde and CAT activity in patients who received general anesthesia in the postoperative period compared with the preoperative period [32]. Zhang et al. and Han et al. demonstrated that propofol exhibits a strong antioxidant effect, attributed to its neuroprotective and cardioprotective properties [33,34]. Lee and Kim showed that propofol increases CAT activity 24 h after surgery and, thus, can increase organ damage [35].

In our study, we observed a significant increase in GST activity at the third measurement in both the GA and SA groups, with no statistically significant differences in the activities of GSH-Px, SOD, GR, and CAT. Evidence suggests that the level of GST expression is a crucial factor in determining the sensitivity of cells to a wide range of toxic chemicals, mainly xenobiotics or products of oxidative stress [36]. However, the increase in GST activity noted in our study can be explained as a transient response of the organism to the administered anesthetic. It cannot be considered to be oxidative stress, as the activity of other antioxidant enzymes is unchanged.

Proteins with the SH (thiol) group are critical compounds in redox-sensitive reactions in plasma [37]. In the GA group, a statistically significant increase in the concentration of total SH groups was observed during surgery (second measurement), indicating an increased cellular redox capacity in this group. Propofol is a lipophilic and phenolic antioxidant that scavenges free radicals, reduces lipid peroxidation, inhibits cellular oxidative damage, and increases tissue glutathione levels [38]. The latter could explain the increased concentration of total SH groups in the GA group during surgery. However, the increase in total SH groups at the second measurement and the decrease to the control level at the third measurement in the GA group in our study may also indicate an increased level of oxidative stress due to delayed analgesia that parturients receive at the moment of clamping the umbilical cord [39]. The transient decrease in non-protein SH groups in the SA group in our study is likely due to surgery, as it returned to control levels two hours after surgery.

In our study, we also tried to examine laboratory parameters and clinical findings that could serve as predictors of oxidative stress. It has been shown that the number of leukocytes, lymphocytes, alanine transaminase, obesity, and thrombophilia can be independent predictors of oxidative stress. Furukawa demonstrated that obesity predicts oxidative stress, which can lead to metabolic syndrome, a finding that aligns with our results [40]. Bogdanović Pristov and her colleagues demonstrated that pregnant women with thrombophilia in the third trimester exhibited significantly higher levels of oxidative stress biomarkers compared to pregnant women without a thrombophilia diagnosis, which is consistent with our findings [41].

Our study has some limitations. First, although the calculated sample size is justified for cohort studies, our study would have benefited from using a larger sample size. The strict inclusion criteria may exclude important clinical scenarios, potentially limiting the applicability of the findings to a broader obstetric population. This study does not provide information on long-term outcomes related to stress or the type of anesthesia. In addition, we did not measure the impact of these outcomes on newborns due to the complexity of obtaining ethics committee approval.

## 5. Conclusions

Changes in the levels of stress hormones, accompanied by changes in glucose, insulin, and thyroid hormone levels, indicate a more pronounced endocrine response in patients treated under general anesthesia compared to those treated with spinal anesthesia, suggesting an advantage of spinal anesthesia in surgical interventions such as cesarean sections. On the other hand, significant changes in the levels of GST and total SH groups over three measurements, without changes in the activities of other antioxidant enzymes, indicate that both types of anesthesia are equally safe in terms of oxidative status in the tissue. In addition, the lymphocyte count was found to be an independent contributor to the change in GST levels across three measurements, which may indicate some changes at the liver level and possibly slower anesthetic metabolism in some patients. All validated predictors should be considered in future studies of this kind.

## Figures and Tables

**Table 1:** The demographic and pregnancy characteristics of participants.

Variable	General Anesthesia Group (n = 51)	Spinal Anesthesia Group (n = 50)	Total	*p*
Age (years)	33.1 ± 0.7	33.2 ± 0.6	33.1 ± 0.46	0.879
Place of residence (%)				0.057
House	27 (52.9)	35 (71.4)	62 (61.4)	
Apartment	24 (47.1)	14 (28.6)	38 (37.6)	
Body mass index (kg/m^2^)	28.6 ± 0.64	28.4 ± 0.56	28.5 ± 0.42	0.734
Body mass index categories (%)				0.828
Underweight	0 (0.0)	0 (0.0)	0 (0.0)	
Normal weight	8 (15.7)	10 (20.0)	18 (17.8)	
Overweight	30 (58.8)	25 (50.0)	55 (54.5)	
Obese	13 (25.5)	15 (30.0)	28 (27.7)	
Waist circumference (cm)	102.7 ± 1.29	101.5 ± 1.04	102.1 ± 0.82	0.482
Smoking status (%)				0.147
No	25 (49.0)	33 (66.0)	58 (57.4)	
Yes	13 (25.5)	6 (12.0)	19 (18.8)	
Ex smoker	13 (25.5)	11 (22.0)	24 (23.8)	
Alcohol consumption (%)				0.989
No	50 (98.0)	49 (98.0)	99 (98.0)	
Yes	1 (2.0)	1 (2.0)	2 (2.0)	
Level of physical activity (%)				0.811
Low	28 (54.9)	28 (56.0)	56 (55.4)	
Medium	22 (43.1)	20 (40.0)	42 (41.6)	
High	1 (2.0)	2 (4.0)	3 (3.0)	
Presence of comorbidity (%)				0.469
No	28 (54.9)	31 (62.0)	59 (58.4)	
Yes	23 (45.1)	19 (38.0)	42 (41.6)	
Type of comorbidity				0.423
Hypertension	2 (3.9)	2 (4.0)	4 (4.0)	
Obesity	10 (19.6)	8 (16.0)	18 (17.8)	
Diabetes	0 (0.0)	1 (2.0)	1 (1.0)	
Insulin resistance	7 (13.7)	6 (12.0)	13 (12.9)	
Asthma	0 (0.0)	2 (4.0)	2 (2.0)	
Hypothireosis	5 (9.8)	9 (18.0)	14 (13.9)	
Thrombophilia	3 (5.9)	4 (8.0)	7 (6.9)	
Allergies (%)				0.796
No	44 (86.3)	44 (88.0)	88 (87.1)	
Yes	7 (13.7)	6 (12.0)	13 (12.9)	
Family history for cardiovascular disease, neurodegenerative or malignant diseases (%)				0.197
Yes	31 (60.8)	24 (48.0)	46 (45.5)	
No	20 (39.2)	26 (52.0)	55 (54.5)	
Previous surgical procedures (%)				0.309
Yes	9 (17.6)	13 (26.0)	22 (21.8)	
No	42 (82.4)	37 (74.0)	79 (79.2)	
Gestational week				0.506
37	1 (2.0)	0 (0.0)	1 (1.0)	
38	6 (11.8)	2 (4.0)	8 (7.9)	
39	24 (47.1)	25 (50.0)	49 (48.5)	
40	18 (35.3)	20 (40.0)	38 (37.6)	
41	2 (3.9)	3 (6.0)	5 (5.0)	

**Table 2:** The results of mixed effects model analysis for predictors of change in oxidative stress parameters over three measurements—list of variables with *p*-value < 0.100 in univariate and 0.05 in multivariate (marked in bold in table) analysis: Glutathione S-transferase (GST); catalase (CAT); glutathione peroxidase (GSH-Px); glutathione reductase (GR); superoxide dismutase (SOD); thyroid-stimulating hormone (TSH); **—*p* < 0.01; *—*p* < 0.05.

GST	CAT	GSH-Px	GR	SOD	SH Groups	Non-Protein SH
BMI Smoking status Neutrophils Lymphocytes * Bicarbonates Potassium C-reactive protein Cortisol Prolactin	Thrombophilia Lymphocytes Potassium Lipase Alanine transaminase TSH	Presence of comorbidity Hypothireosis Thrombophilia Alanine transaminase * TSH	Omega-3 supplementation Obesity ** Thrombophilia ** Leukocytes * Aspartate transferase	Folic acid supplementation Magnesium supplementation Potassium	Place of residence Comorbid allergies Hemoglobin Hematocrit Neutrophils Leukocytes	Hypothireosis Thrombophilia Lipase Insulin

## Data Availability

Data are available at https://hdl.handle.net/21.15107/rcub_ibiss_7499 (accessed on 21 July 2025).

## Supplementary Materials

The following supporting information can be downloaded at https://www.mdpi.com/article/10.3390/life15081158/s1. Table S1. Blood count and biochemical parameters in general and spinal anesthesia over three measurements: 1—one hour before the start of the cesarean section, 2—at the moment of clamping the umbilical cord, 3—two hours after the end of the cesarean section.
