# Supplementary material for: Physiological and Oxidative Stress in General and Spinal Anesthesia for Elective Cesarean Section in Women: Is There Any Difference?
Source: Life (Basel). 2025 Jul 22;15(8):1158. doi: 10.3390/life15081158 (PMC12387363; doi:10.3390/life15081158)
Supplement: Supplementary file 1 [file life-15-01158-s001.zip › life-3732809-supplementary.pdf]

Table S1. Blood count and biochemical parameters in general and spinal anesthesia over three measurements: 1 – one hour before the start of the cesarean section, 2 – at the moment of clamping the umbilical cord, 3 – two hours after the end of the cesarean section

variable	General anesthesia			Spinal anesthesia		
	**1**	**2**	**3**	**1**	**2**	**3**
erythrocytes	3.95 (0.37)	3.78 (3.22)	3.65 (0.39)	4.08 (0.38)	3.69 (0.33)	3.71 (0.31)
thrombocytes	223.04 (68.92)	219.67 (71.75)	207.96 (64.26)	223.62 (42.51)	200.84 (41.56)	197.36 (35.58)
leukocytes	9.90 (2.40)	11.22 (2.55)	15.15 (4.13)	9.62 (2.98)	9.77 (2.31)	11.22 (2.83)
neutrophils	71.15 (7.00)	69.84 (7.13)	85.65 (4.84)	71.79 (10.34)	81.67 (85.66)	80.97 (5.37)
basophils	0.39 (0.27)	0.33 (0.16)	0.22 (0.11)	0.41 (0.21)	0.41 (0.20)	0.28 (0.13)
lymphocytes	20.07 (5.78)	21.71 (5.75)	8.91 (3.74)	21.81 (25.92)	21.61 (6.20)	12.48 (4.45)
tryglicerides	2.90 (1.01)	3.07 (0.94)	2.58 (0.95)	2.89 (1.03)	2.54 (0.78)	2.45 (0.81)
hemoglobin	117.54 (11.26)	112.92 (10.33)	108.94 (11.49)	122.12 (8.20)	110.51 (8.03)	111.34 (8.31)
hematocrit	0.36 (0.03)	0.34 (0.03)	0.33 (0.03)	0.37 (0.03)	0.33 (0.02)	0.34 (0.02)
fibrinogen	4.14 (0.84)	3.84 (0.70)	3.51 (0.65)	4.08 (0.62)	3.54 (0.50)	3.40 (0.45)
creatinine	51.02 (10.75)	48.84 (9.90)	50.62 (8.21)	46.56 (6.23)	49.18 (7.14)	46.22 (6.77)
albumins	35.26 (2.51)	32.58 (1.98)	30.34 (2.72)	36.76 (2.07)	31.49 (1.93)	30.88 (2.13)
C-reactive protein	7.80 (8.14)	7.16 (6.10)	7.13 (5.68)	3.93 (2.59)	4.83 (5.21)	4.78 (4.97)
bicarbonates	19.86 (2.38)	19.14 (2.84)	18.94 (2.37)	19.02 (2.15)	17.96 (1.79)	18.58 (1.70)
chloride	105.86 (2.15)	107.37 (2.18)	106.76 (2.45)	106.30 (2.98)	108.31 (2.75)	107.82 (2.42)
potassium	4.13 (0.24)	3.85 (0.26)	4.17 (0.34)	4.19 (0.26)	3.90 (0.29)	3.99 (0.29)
alanine transaminase	12.28 (5.07)	11.04 (3.52)	11.06 (4.74)	15.48 (8.60)	13.63 (8.37)	13.50 (7.68)
aspartate transferase	19.20 (4.57)	18.12 (3.98)	20.94 (5.14)	22.66 (7.92)	20.76 (7.64)	22.22 (7.55)
lipase	55.24 (50.95)	46.27 (47.21)	42.69 (41.26)	78.90 (55.38)	73.94 (56.95)	78.30 (60.64)
